# A longitudinal analysis of patient satisfaction with care and quality of life in ambulatory oncology based on the OUT-PATSAT35 questionnaire

**DOI:** 10.1186/1471-2407-14-42

**Published:** 2014-01-25

**Authors:** Thanh Vân France Nguyen, Amélie Anota, Anne Brédart, Alain Monnier, Jean-François Bosset, Mariette Mercier

**Affiliations:** 1 Radiotherapy Department, Besançon University Hospital, Besançon 25030, France; 2 Radiotherapy Department, Montbéliard Hospital, Montbéliard 25200, France; 3 Clinical Research Department, EA 3181, Université de Franche Comté, Besançon 25030, France; 4 Psycho-Oncology Unit, Institut Curie, Paris cedex 75 231, France; 5 Health-Related Quality of Life in Oncology Platform, Cancéropole Grand Est, Strasbourg University Hospital Haute Pierre, Strasbourg 67098, France; 6 Radiotherapy Department, Gustave Roussy Cancer Center, 114 Rue Edouard Vaillant, 94805 Cedex Villejuif, France

**Keywords:** Patient satisfaction, Determinants of satisfaction, Quality of life, Ambulatory oncology

## Abstract

**Background:**

In the oncology setting, there has been increasing interest in evaluating treatment outcomes in terms of quality of life and patient satisfaction. The aim of our study was to investigate the determinants of patient satisfaction, especially the relationship between quality of life and satisfaction with care and their changes over time, in curative treatment of cancer outpatients.

**Methods:**

Patients undergoing ambulatory chemotherapy or radiotherapy in two centers in France were invited to complete the OUT-PATSAT35, at the beginning of treatment, at the end of treatment, and three months after treatment. This questionnaire evaluates patients’ perception of doctors and nurses, as well as other aspects of care organization and services. Additionally, for each patient, socio-demographic and clinical characteristics, and self-reported quality of life data (EORTC QLQ-C30) were collected.

**Results:**

Of the 691 patients initially included, 561 answered the assessment at all three time points. By cross-sectional analysis, at the end of the treatment, patients who experienced a deterioration of their global health reported less satisfaction on most scales (p ≤ 0.001). Three months after treatment, the same patients had lower satisfaction scores only in the evaluation of doctors (p ≤ 0.002). Furthermore, longitudinal analysis showed a significant relationship between a deterioration in global health and a decrease in satisfaction with their doctor and, conversely, between an improvement in global health and an increase in satisfaction on the overall satisfaction scale. Global health at baseline was largely and significantly associated with all satisfaction scores measured at the following assessment time points (p < 0.0001). Younger age (<55 years), radiotherapy (versus chemotherapy) and head and neck cancer (versus other localizations) were clinical factors significantly associated with less satisfaction on most scales evaluating doctors.

**Conclusions:**

Pre-treatment self-evaluated global health was found to be the major determinant of patient satisfaction with care. The subsequent deterioration of global health, during and after treatment, emphasized the decrease in satisfaction scores, mainly in the evaluation of doctors. Early initiatives aimed at improving the delivery of care in patients with poor health status should lead to improved perception of the quality of care received.

## Background

In the last few decades, patient satisfaction has become an important endpoint in the assessment of the quality of care, which is increasingly required by accreditation agencies in monitoring of quality of hospital care. Moreover, satisfaction with care may influence patient compliance to treatment and consequently, impact on disease outcome.

In the setting of oncology, advances in diagnostics, treatment, supportive care and rehabilitation call for regular evaluations, in order to determine whether patients’ expectations are being met by the complex and multidisciplinary nature of the healthcare that they receive. Thus, patient satisfaction surveys can help to identify patient groups who merit additional attention or even targeted interventions, and can also highlight areas of the care process where there is room for improvement. Several studies have focused on patient satisfaction in specific cancer treatments, such as gastro-esophageal [1], breast [2,3], colorectal [4], and gynaecological cancers [5]. However, few included a large sample size and a heterogeneous population as regards cancer types [6-8]. Similarly, a number of studies have been conducted to evaluate the predictors of patient satisfaction in various oncology care settings, and more recently in the context of ambulatory treatment [9-12]. Socio-demographic characteristics (age, sex, education level, marital status) and health status are the most widely studied predictors of satisfaction [7,8,13-15], but conflicting results have been reported, especially regarding the relationship between self-perceived quality of life (QoL) and satisfaction with care [16-20]. However, to the best of our knowledge, no study has yet evaluated the effect of longitudinal changes in a patient’s QoL on their satisfaction with care over time.

In a previous cross-sectional study of French cancer outpatients evaluated at the beginning of their ambulatory chemo- or radiotherapy, we identified a number of clinical factors (primary cancer, type of treatment received) and socio-demographic factors (marital status, age) that were significantly associated with different domains of satisfaction with care. Nevertheless, the major determinant was the patient’s global health status, suggesting that self-reported QoL is a key element in understanding cancer patient satisfaction [21].

The goal of the present study was, firstly, to ascertain the influence of clinical and socio-demographic factors previously identified as potential determinants of satisfaction with care, at several assessment time points during and after treatment. Secondarily, we investigated the influence of longitudinal changes in self-reported QoL on variations in satisfaction with care, as measured by multi-dimensional questionnaires.

### Methods

We conducted a multicenter, prospective cohort study of cancer outpatients from the beginning of treatment and until three months after the end of treatment.

The protocol was approved by the regional ethics committee of the University Hospital of Besançon for both participating hospitals (Comité de Protection des Personnes Est-II, France), the National French Data Protection Agency, and was supported by a regional grant from the French National Hospital Research Programme (Programme Hospitalier de Recherche Clinique, PHRC). All patients provided written informed consent.

#### Patients

Patients were enrolled in two centers (one university teaching hospital and one non-academic hospital) in eastern France between January 2005 and December 2006. Inclusion criteria were: patients aged over 18 years, able to understand written and spoken French, able to provide written consent, able to complete the questionnaires, with a confirmed histological diagnosis of cancer, and due to undergo ambulatory treatment by chemo- or radiotherapy.

The following cancers were included in 9 treatment groups: 2 prostate cancer groups (radiotherapy only or surgery followed by radiotherapy), 3 breast cancer groups (surgery plus radiotherapy, or surgery plus chemo and radiotherapy, or chemotherapy alone), 2 head and neck cancer groups (surgery plus radiotherapy or radiotherapy with or without concurrent chemotherapy), 1 rectum cancer group (radiochemotherapy plus surgery) and 1 lung cancer group (chemo and radiotherapy).

#### Study procedures and measures

As described previously [21], patients were orally invited to participate in the study by the research technician when they came to the hospital at the beginning of the first week of radiotherapy or at the first cycle of chemotherapy. However, it was not technically possible to meet all patients on a systematic basis. Once the patient agreed to participate and provided informed consent, the socio-demographic questionnaire was completed with the research technician. Patients were asked to complete the EORTC QLQ-C30 and OUT-PATSAT35 questionnaires at three different time points: i.e. at the beginning of treatment (at the end of the first week of radiotherapy or at the second cycle of chemotherapy), at the end of treatment (at the last week of radiotherapy or at the sixth cycle of chemotherapy) and three months after the end of treatment. Only one treatment group, namely breast cancer patients who underwent surgery plus chemotherapy and radiotherapy, had to complete the questionnaires at four different time points because we assessed their satisfaction with care both at the end of chemotherapy and at the end of radiotherapy. The questionnaire for the first time point was completed at the hospital. For the two subsequent time points (end of treatment and three months after treatment), questionnaires were given to the patient during treatment visits or consultations, and were completed at home and mailed back using a stamped addressed envelope. If necessary, patients were called by phone and reminded to return the questionnaires two weeks later.

The EORTC IN-PATSAT32 questionnaire was developed by the EORTC QOL group in order to assess patient satisfaction with care in oncology hospitals [22]. The OUT-PATSAT35 questionnaire was adapted from IN-PATSAT32 for use among outpatients treated by ambulatory chemotherapy or radiotherapy. Adequate psychometric properties have been reported for the French and Spanish language versions [23-25].

The OUT-PATSAT35 questionnaires contains 35 items covering 12 multi-item scales organized into three sections of four scales each: two sections evaluating doctors and nurses (for chemotherapy) or radiation therapists (for radiotherapy), as regards their technical skills (knowledge, experience, assessment of physical symptoms), interpersonal skills (interest, willingness to listen), provision of information (about the disease, medical tests and treatment), and availability (time devoted to patients). The third section evaluates the organization of the department, the exchange of information between caregivers (coherence, identification of the reference doctor), the interpersonal skills and quality of information provided by other hospital staff, waiting times (for consultation, medical tests, or treatment), the physical environment (access, comfort, orientation), and lastly, a single-item : the overall satisfaction scale.

Items are rated on a 5-level Likert scale as follows: “poor”, “fair”, “good”, “very good”, “excellent”. All scores are linearly transformed on a 0 to 100 scale, with a higher score reflecting a higher level of satisfaction.

The EORTC QLQ-C30 (version 3.0) is a 30-item self-assessment questionnaires of QoL comprising 5 functional scales (physical, role, emotional, cognitive, social), 9 symptom scales (fatigue, nausea or vomiting, pain, dyspnea, insomnia, constipation, diarrhea, financial difficulties) and finally, a global health scale.

The assessment of socio-demographic and disease-related variables has been described in detail elsewhere [21].

#### Statistical methods

Characteristics of patients who completed the whole questionnaire were compared to those who returned questionnaires with missing data using Fisher’s exact test, or the Chi-square test for categorical data, and the Student t test for continuous data. If missing data in questionnaires were identified as MNAR (missing not at random), each scale score was estimated with a multiple imputation by the Markov Chain Monte Carlo method (MI procedure).

To illustrate the clinical significance of the variation in QoL, the within-group changes in EORTC QLQ-C30 scores (follow-up scores minus baseline) were categorized in three classes, improvement, no change, or deterioration, with a minimal difference, of either 5 points or 10 points, defining a little change or a moderate change respectively [26]. This method was used for each EORTC QLQ-C30 scale. Furthermore, because we hypothesized that the variation in QoL would not be linear (worse at the end of treatment compared to baseline, due to treatment side effects, and probably better three months after, if recovery had been possible), two models were built using the QoL score changes: i.e. between the beginning and the end of treatment on the one hand, and between the beginning of treatment and three months after the end of treatment on the other hand.

Significant categorical variables (primary localization, type of treatment, age, sex, marital status, leisure activities, home-hospital distance and monthly income) and continuous variables (EORTC QLQ-C30 scores at baseline and changes in global health, emotional functioning, social functioning, sleep, fatigue and pain) identified as possible predictors in previous analysis at baseline [21], were introduced into the multivariate models. However, primary localization (breast, prostate, head and neck, rectum, or lung) and type of treatment received (radiotherapy and/or chemotherapy) were included into two separate models due to the strong colinearity between these two variables. A general linear model (analysis of variance), taking into account repeated measures for a same patient, was used for each scale score of the OUT-PATSAT35 questionnaire at the three assessment time points. Interaction with time for each variable was also investigated.

We distinguished two levels of analysis of satisfaction scores: first, a longitudinal analysis evaluating within-group changes between two assessment time points (follow-up scores at the end of treatment minus baseline and follow-up scores at three months after the end of treatment minus baseline), and secondly a cross-sectional analysis evaluating between-group changes at the end of treatment and three months later.

The significance level for multivariate analysis was set at α = 0.005 (according to Bonferroni correction for multiple testing). All tests were two-sided.

For the interpretation of the satisfaction scores, we considered the minimal difference defined as clinically meaningful by Osoba *et al.,* namely a mean change of at least 5 points [26].

Statistical analysis was performed using Statistical Analysis Software (version 9.1, SAS Institute, Cary, NC, USA).

## Results

### Patient characteristics at baseline

733 patients met the eligibility criteria and were invited to participate in the study: 42 patients (5.7%) declined, and the remaining 691 (94.3%) patients were included. Baseline characteristics of the study population are summarized in Table 1. Median age was 65 years (range 29–88), with a balanced proportion of men and women. All patients were treated with curative intent except for 5 patients who had metastases and were treated by chemotherapy alone. Based on the hypothesis that these patients could behave differently from the rest of the patients, we excluded them from subsequent analyses. Most cancer localizations treated were breast (44%) and prostate cancer (31%).

**Table 1:** Socio-demographic and clinical characteristics of the study population

**Characteristics**		**Number of patients (%)**
**Center**	Teaching hospital	382 (55.3)
	Local hospital	309 (44.7)
**Sex**	Male	356 (51.5)
	Female	335 (48.5)
**Age**	Median [min; max]	65 [29;88]
	30-55	152 (22)
	56-65	186 (26.9)
	66-75	255 (36.9)
	76-88	98 (14.2)
**Marital status**	Single or separated	136 (19.9)
	Living with partner, or family	548 (80.1)
**Education level**	Primary	317 (47.1)
	Secondary	167 (24.8)
	High school diploma or higher	189 (28.1)
**Employment status**	Employed	175 (25.6)
	Retired or unemployed	508 (74.4)
**Monthly income in Euro**	<MW	70 (11.2)
	MW- 1499	218 (34.8)
	1500 -2999	247 (39.4)
	≥ 3000	92 (14.7)
**Localization treated**	Prostate RT	128 (18.5)
	Surgery + RT	82 (11.9)
	Head and neck Surgery + RT	22 (3.2)
	RT+/-CT	71 (10.3)
	Breast Surgery + RT	205 (29.7)
	Surgery + CT + RT	100 (14.5)
	CT	5 (0.7)
	Rectum RT + CT + surgery	23 (3.3)
	Lung CT + RT	55 (8)
**Chemotherapy**	Yes	221 (32)
**Radiotherapy**	Yes	585 (84.7)

Min = minimum; max = maximum; MW = minimum wage; RT = radiotherapy, CT = chemotherapy.

### Missing data on the OUT-PATSAT35 questionnaires

Missing data in questionnaires were identified as MNAR, so each scale score was estimated with a multiple imputation by age and treatment type (radiotherapy vs. chemotherapy).

Six patients were non-responders at baseline and were excluded from the analysis. 49 patients responded at baseline only, and 79 patients responded at the beginning and at the end of treatment but not at 3 months after treatment. Comparison of characteristics between these 128 patients lost to follow-up (19% of the all those included) and the 561 patients who answered at all 3 assessment time points revealed a lower level of QoL at baseline and a higher frequency of lung or head and neck cancers.

### Variation of satisfaction and QoL scores

**Figure 1:**
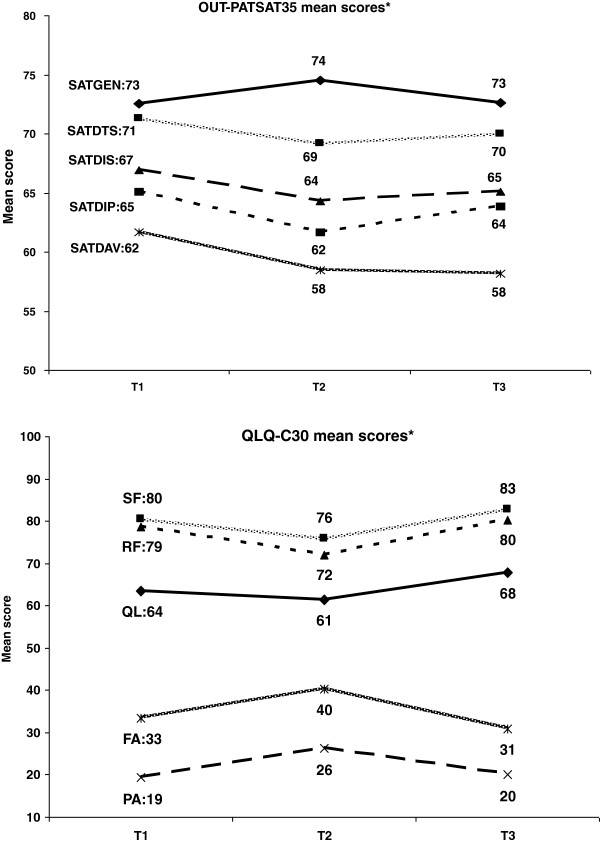
Longitudinal evolution of OUT-PATSAT35 and QLQC30 scores at the 3 assessment time points*. *Only the most representative dimensions are shown. Abbreviations: SATGEN = overall satisfaction, SATDTS doctor technical skills, SATDIS = doctor interpersonal skills, SATDIP = information provided by doctor, SATDAV = doctor availability, SF = social functioning, RF = role functioning, QL = global health, FA = fatigue, PA = pain. T1: start of treatment, T2 : end of treatment, T3 : three months after the end of treatment.

Mean satisfaction scores by scale did not change much over time whereas mean EORTC QLQ-C30 scores deteriorated between baseline and the end of treatment, then improved three months after treatment (Figure 1).

### Multivariate analysis of satisfaction scores

The longitudinal analysis showed significant relationships between the within-group changes in global health score (adjusted for global health score at baseline), and the within-group changes in satisfaction scores. During the treatment period, an improvement in the global health score, of a minimum of either 5 or 10 points, was significantly related to an increase in overall satisfaction score (mean score differences of 9 and 12 points respectively in the model with radiotherapy, p ≤ 0.0002, Additional file 1).

Between the beginning of treatment and three months after the end of treatment, an improvement in global health, with a minimal difference of either 5 or 10 points, was again significantly related to an increase in overall satisfaction score (mean score difference of 7 points, in both cases, in the model with radiotherapy, p ≤ 0.004), whereas a deterioration in global health, with a minimal difference of either 5 or 10 points, was significantly linked to a decrease in satisfaction with doctors’ technical skills, interpersonal skills, and provision of information (mean score differences ranged from 8 to 16 points in the model with primary localization, p ≤ 0.0001, Additional file 1).

By cross-sectional analysis evaluating between-group changes in satisfaction scores, at the end of the treatment, patients who reported a deterioration in global health during treatment were significantly less satisfied on most scales, as compared to those who reported an improvement in global health (mean score differences ranged from 8 to 20 points). The greatest differences in satisfaction scores were observed in overall satisfaction, in satisfaction with doctors’ technical skills, interpersonal skills, provision of information and nurses or radiation therapists’ provision of information and availability, and after adjustment for a minimal change in global health of 10 points compared to a minimal change of 5 points (p ≤ 0.001, Additional file 2).

Three months after the end of treatment, patients who experienced a decrease in global health scores since the start of treatment reported less satisfaction mainly with doctors’ technical skills, interpersonal skills, provision of information and availability (mean score differences ranged from 10 to 19 points). This effect was again more significant after adjustment for a minimal change in global health of 10 points compared to a minimal change of 5 points (p ≤ 0.002, Additional file 2).

As reported in previous work [21], perceived global health at the beginning of treatment remained largely and significantly associated with all satisfaction scores, not only at the start of treatment but also at the subsequent time points (p < 0.0001, data not shown)

**Figure 2:**
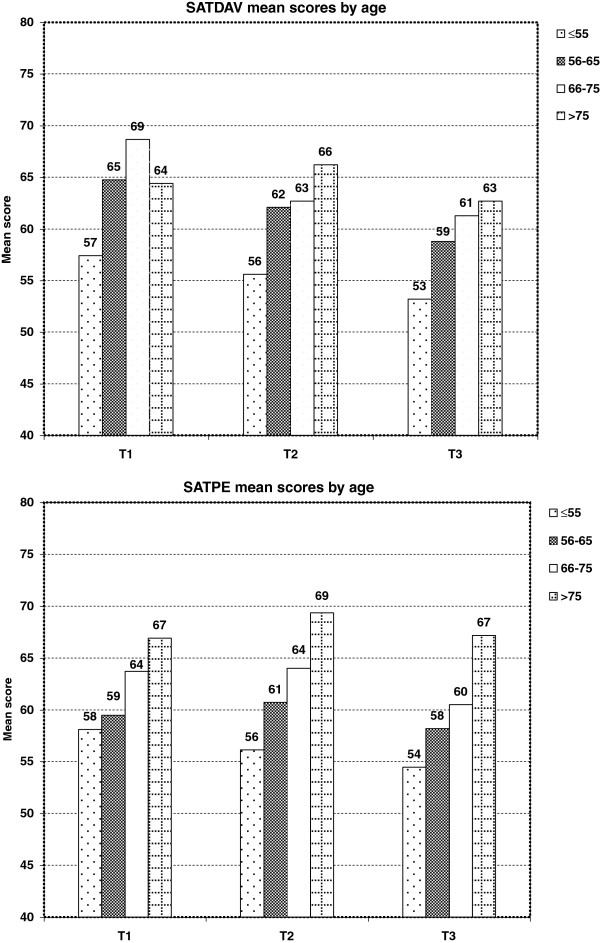
**Influence of age on satisfaction scores at the 3 assessment time points**^**$**^**.**^$^Mean satisfaction scores adjusted for radiotherapy, age, marital status, centre, level of global health at inclusion and minimal difference scores of 10 points for global health changes. Abbreviations: SATDAV = doctors’ availability, SATPE = physical environment.

Younger age (≤55 years) was significantly linked to dissatisfaction with doctors’ availability and the hospital environment at the three assessment time points (p ≤ 0.002): mean score differences, between patients ≤55 years old and those >75 years old, were ≥10 points at the end after treatment and three months later (Figure 2).

**Figure 3:**
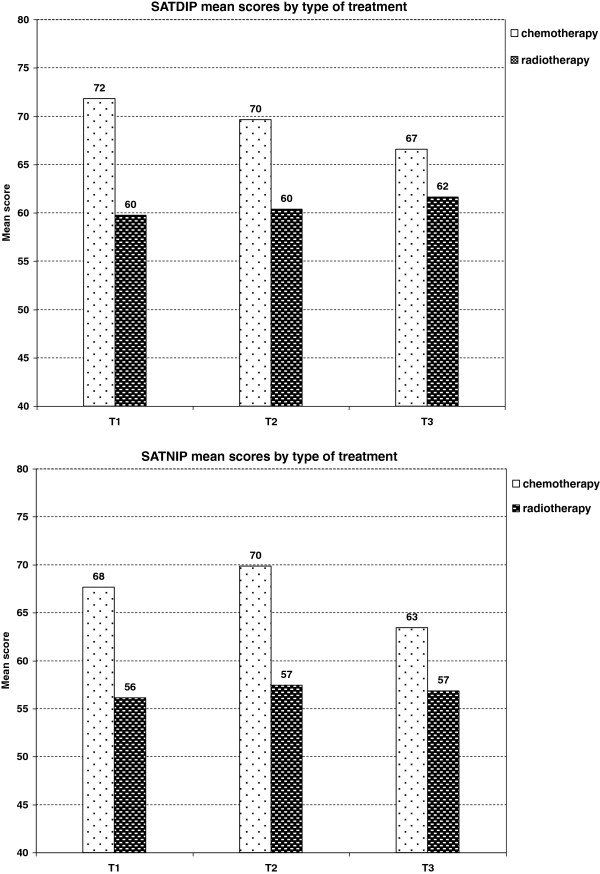
**Influence of type of treatment on satisfaction scores at the 3 assessment time points**^**$**^**.**^$^Mean satisfaction scores adjusted for radiotherapy, age, marital status, centre, level of global health at inclusion and minimal difference scores of 10 points for global health changes. Abbreviations: SATDIP = doctors’ information provision, SATNIP = nurses’ information provision.

Radiotherapy (as opposed to chemotherapy) was significantly associated with lower satisfaction with doctors’ technical skills, interpersonal skills, doctors’ and nurses’ provision of information and waiting time (p ≤ 0.005). The score difference was larger at the end of treatment than three months later (for instance, mean score differences of 10 and 5 points respectively in satisfaction with doctors’ provision of information, Figure 3).

**Figure 4:**
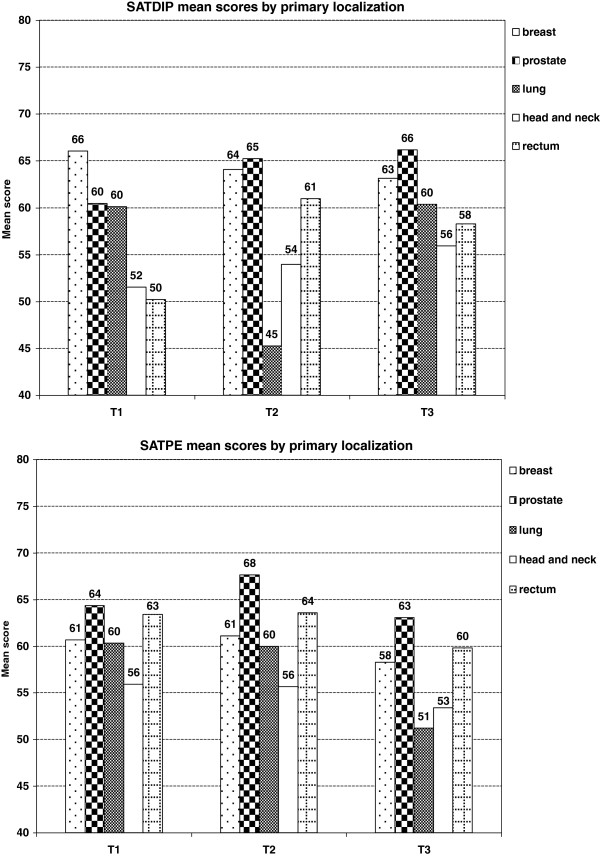
**Influence of primary localization on satisfaction scores at the 3 assessment time points**^**$**^**.**^$^Mean satisfaction scores adjusted for primary localization, age, marital status, centre, level of global health at inclusion and minimal difference scores of 10 points for global health changes. Abbreviations: SATDIP = doctors’ information provision, SATPE = physical environment.

Patients treated for head and neck cancer were less satisfied with doctors’ provision of information, and hospital environment as compared to those treated for prostate cancer at the three assessment time points: for instance, mean score differences ranged from 8 to 11 points in satisfaction with doctors’ provision of information (p = 0.002, Figure 4).

## Discussion

Initial self-reported global health and its variation across time were found to be the major determinants of patient satisfaction with ambulatory care during and after chemotherapy or radiation therapy. The longitudinal analysis studying within-group changes in satisfaction scores, showed a different effect of changes in global health score, depending on the satisfaction scale considered: an improvement in global health was significantly related to an increase in the overall satisfaction score, whereas a deterioration in global health was linked to a decrease in satisfaction with doctors, this latter relationship becoming significant during the period between the beginning of treatment and three months after the end of treatment. The cross-sectional analysis (evaluating between-group changes in satisfaction scores) showed lower satisfaction scores in patients who experienced a deterioration in their global health on most scales of OUT-PATSAT35 at the end of treatment, and mainly on the scales reporting satisfaction with doctors three months after the end of treatment.

In the longitudinal analysis of satisfaction scores, we observed discrepancies in the results between the overall satisfaction scale and the doctors’ scales. These findings may be explained by the limited score variability due to the ceiling effect that was frequently observed on the overall satisfaction scale, where a large proportion of patients scored the maximum [27]. In other words, high satisfaction ratings do not necessarily mean that the patients had had a positive experience of healthcare. Conversely, dissatisfaction rates may better reflect a minimum level of negative experience with healthcare [28]. In our study, the deterioration of patient QoL during treatment, probably linked to acute toxicities, may have increased anxiety related to the cancer issue or the potential complications of the treatment. Thus, it may have generated greater patient expectations about medical information, making them more difficult to satisfy. In a large outpatient cohort of more than 4600 cancer patients, Feyer et al. [29] showed that fatigue and the number of treatment side effects self- reported by the patient had a negative impact on the patients’ assessment of cancer care: more than 30% of these patients were not satisfied with the information they received about adverse events and their handling. Therefore, the question remains whether a reduction in these side-effects would result firstly in better patient QoL, and subsequently, in improved satisfaction with care. Future research should include a thorough evaluation of cancer-related adverse events using a validated instrument that systematically assess both the presence and the severity of symptoms.

Furthermore, it is noteworthy that the greatest decrease in satisfaction scores was observed in the scales evaluating doctors compared to nurses or radiation therapists, and especially when considering the assessment at three months after the end of treatment. It can thus be hypothesized that during this period, patients have had greater expectations with doctors than with other caregivers, essentially linked to a need of information about the effectiveness of treatment and the cancer prognosis.

In our study, radiotherapy, as compared to chemotherapy, was significantly linked to lower satisfaction scores in terms of information provided and waiting times. These results should be interpreted with caution, however, because the few patients receiving chemotherapy were those treated for breast cancer, and in these patients, the end of chemotherapy was not the end of the overall treatment as they had radiotherapy afterwards. Thus, the expectations of these patients vis-à-vis their caregivers may not have been as high as those in whom the end of radiotherapy was also the end of their treatment.

Stiegelis *et al.* analyzed the psychological functioning of patients treated with radiotherapy through 45 studies [30] and found no significant differences in feelings of anxiety, depressive symptoms or psychological distress between patients treated with radiotherapy and those treated with other treatment modalities. Furthermore, this body of work suggests a strong relationship between the amount of physical side-effects of treatment and psychological dysfunction, which tends to be higher in the last week of radiotherapy when adverse events reach their peak. During the months following the completion of treatment, psychological dysfunction may also continue. A possible reason for this finding is that cancer patients may enter a period in which the persistence of treatment side effects associated with the uncertainty about the effectiveness of radiotherapy in controlling the cancer, on the one hand, and the loss of the support network (i.e. relations with medical staff), on the other hand, combine to create a difficult context that can promote psychological distress, and may be a cause of dissatisfaction with the information supplied by care providers [31].

Patients suffering from head and neck cancer, who were the least satisfied with the information provided by doctors and the hospital environment, are probably those who experience the most symptoms and physical side-effects, either associated with their illness or the radiation therapy, involving impairments in functional domains as eating, speaking or breathing. Moreover, the treatment toxicities are often intense, last for several months, even persisting as long-term sequelae. An English study assessing satisfaction with the provided information to 82 head and neck cancer patients, in which 73% received radiotherapy, revealed a need for more information about the impact of treatment and especially the long-term effects [32]. Regarding satisfaction between different primary cancers, in a national survey of cancer patients’ satisfaction with care in 55,674 English patients, Sherlaw-Johnson et al. found that hospital satisfaction varied by cancer type (for breast, colorectal, lung and prostate cancer patients): breast and lung cancer patients were more satisfied than patients with colorectal cancer, while prostate cancer patients tended to be least satisfied [6]. Conversely, in a Canadian study including 2,790 patients, Sandoval et al. found that prostate cancer patients reported a higher level of overall satisfaction compared to other primary localizations, including head and neck, brain, breast, gynaecological, lung and digestive cancers [7].

Consistent with a number of previous studies [6,8,15], we show that younger age was linked to less satisfaction with some aspects of care. It has been suggested that older people trust their doctor more and have more modest expectations [27].

The overall response rate in our study population was in line with the methodological minimum requirement of 80%, since 99% of patients completed the OUT-PATSAT35 questionnaires at inclusion, 88% at the second assessment and 81% at the third time point, confirming the acceptability of the OUT-PATSAT35 questionnaire in a large outpatient sample. In satisfaction surveys, response rates have been shown to range from 66 to 77%, depending on the procedure for data collection [33].

Patients with a lower QoL at baseline and treated for lung or head and neck cancers were less likely to respond to the follow-up assessments. Despite this potential bias, in our longitudinal analysis, we identified a significant relationship between a low global health score at baseline and low satisfaction scores in the subsequent assessments. It can thus be hypothesized that this effect of initial global health may have been underestimated.

Another potential bias related to the study procedures has to be underscored. Since the questionnaires at the second and the third time points were fulfilled at home, patients’ responses could have been influenced by their proxies. Sandoval et al. shown that scores evaluating overall perception of the quality of care were significantly lower in cases where someone other than the patient completed the survey [7].

Although a number of variables were found to be significantly associated with satisfaction scores, all the combined covariates explained only 10 to 12% of the observed variation in satisfaction with care in our sample. Having the disease under control as an outcome of care was not introduced into the model, since this criterion was considered not to be relevant in the study context. Most patients underwent surgery and had no residual disease, and for patients who did not undergo surgery, a three month delay was insufficient to ascertain whether the cancer was under control. In a sample of the World Health Survey for 2003 including 16,384 patients in 21 European Union countries, factors such as age, income, education, immunization coverage, and self-reported health status were significantly associated with satisfaction with the healthcare system. However, these predictors only explained 7.5% of the variation in satisfaction. When factors linked to patient experience with care were added to the model, the proportion of variation in satisfaction scores explained only increased to 17.5%. The authors suggest that some of the remaining variation in satisfaction with the healthcare system could be explained by broader societal factors, like the media’s influence on patients’ perception of the health system, but such factors are unfortunately difficult to capture with questionnaires [15].

Despite this gap in our understanding of the factors related to the patient’s determination of their satisfaction with care, one of the main motivations for measuring patient satisfaction is to help recognize and thus resolve potential patient dissatisfaction problems. From a management perspective, it may contribute to emphasising priorities in terms of investments for health care organizations: for example, in our study, waiting times (for obtaining an appointment or how easy it was to reach a caregiver by phone) and the physical environment of the hospital (access, comfort) were the domains in which patients reported lowest levels of satisfaction. Thus, these findings could support a request for more human resources on the one hand, and a project to extend parking capacity on the other hand. From a health care provider’s perspective, satisfaction surveys aim to identify patients’ needs and expectations. In our study, dissatisfaction was reported on the scale relating to the doctors’ provision of information, especially in patients younger than 55 years and those treated for head and neck cancer. As a result, concerted efforts to deliver adequate information adapted to these patients’ groups had to be made. Effective doctor-patient communication has been associated with improved psychological functioning of the patient, adherence to treatment, higher QoL and greater satisfaction with care [34-36]. However, with cancer patients, doctors are often confronted with difficult issues for which they are unprepared, such as communicating bad news, preparing for adverse procedures, exploring treatment options, enrolling the patient in clinical trials or discussing prognosis. Consequently, various initiatives to improve communication between doctors and cancer patients have been developed, focusing either on patients (standardised information provided on a video or in a medical information package [37,38]) or on doctors (training in communication skills [39,40]).

Our results showed that patient’s global health at the beginning of treatment was the major determinant of satisfaction with care, during and after treatment. It can be hypothesized that identifying these patients with poor QoL as early as possible in the care pathway, and providing them with comprehensive supportive care should improve their perception of the subsequent delivery of the cancer treatment. It has been shown that the measurement of individual patient health-related QoL can be used in clinical practice to detect physical or psychological problems providing useful information to caregivers and thus, facilitating doctor-patient communication [41,42]. Furthermore, previous work by Velikova et al. indicates that routine repeated QoL measurements with feedback of results to doctors leads to an increased discussion of health-related QoL issues, resulting in clinically meaningful improvement in patient well-being [43]. In addition, according to a National Institutes of Health expert panel report, clinicians should routinely use brief assessment tools to ask patients systematically about symptoms and side-effects, with the aim of initiating appropriate treatment as soon as possible [44].

Lastly, patient satisfaction with care reflects the extent to which the patients’ needs, desires or preferences are met. In order to satisfy the patients’ expectations, healthcare providers should adapt their behavior and communication style to each individual, and thus move towards patient-centered delivery of care.

## Conclusions

Our study brought to light the major impact of self-reported overall health status at the beginning of the cancer treatment, as well as its variations over time, on most domains of satisfaction with care. To the best of our knowledge, this is the first longitudinal study to report on the responsiveness of a satisfaction questionnaire to QoL changes over time.

The strong relationship between health-related overall QoL and patient satisfaction with care underscores the necessity of evaluating these two subjective measures when comparing different treatments, patterns of care, or health care systems at a given time point, but also when assessing variations in patient satisfaction over time with intent to measure, for instance, the health system “responsiveness” in improving the quality of care.

From the healthcare professional’s point of view, it might be expected that early initiatives aimed at improving the delivery of care in patients with poor health status would lead to a better perception of the quality of care received and thus a higher satisfaction with care.

Challenges in the future should address the appropriate interpretation, report and use patient satisfaction survey data to efficiently improve quality of care.

## Competing interest

The authors declare that there is no conflict of interest.

## Authors’ contributions

TVFN participated to the coordination of the study, performed the statistical analysis, the interpretation of data and drafted the manuscript. AA helped to the statistical analysis and the interpretation of data. AB revised the manuscript critically for important intellectual content. JFB and AM participated in the conception and design of the study. MM participated in the conception and coordination of the study, helped to the statistical analysis and the interpretation of data. All authors read and approved the final manuscript.

## Pre-publication history

The pre-publication history for this paper can be accessed here:

http://www.biomedcentral.com/1471-2407/14/42/prepub

## Supplementary Material

Additional file 1 Longitudinal analysis of satisfaction scores at T2 and T3 assessments by QoL changes. Click here for file

Additional file 2 Cross-sectional analysis of satisfaction scores at T2 and T3 assessments by QoL changes. Click here for file
