# Supplementary material for: A longitudinal analysis of patient satisfaction with care and quality of life in ambulatory oncology based on the OUT-PATSAT35 questionnaire
Source: BMC Cancer. 2014 Jan 25;14:42. doi: 10.1186/1471-2407-14-42 (PMC3922727; doi:10.1186/1471-2407-14-42)
Supplement: Additional file 1 — Longitudinal analysis of satisfaction scores at T2 and T3 assessments by QoL changes. [file 1471-2407-14-42-S1.doc]

Additional file 1: Longitudinal analysis of satisfaction scores at T2 and T3 assessments by QoL changes

| Mean differences of Sat35 (period T1-T2) | | SATGEN | | | SATDTS | | | SATDIS | | | SATDIP | | | SATNTS | | | SATNIS | | | SATNIP | | |
| --- | --- | --- | --- | --- | --- | --- | --- | --- | --- | --- | --- | --- | --- | --- | --- | --- | --- | --- | --- | --- | --- | --- |
|  |  | T1^$^  (SD) | T2-T1* | T2-T1** | T1^$^  (SD) | T2-T1* | T2-T1** | T1^$^  (SD) | T2-T1* | T2-T1** | T1^$^  (SD) | T2-T1* | T2-T1** | T1^$^  (SD) | T2-T1* | T2-T1** | T1^$^  (SD) | T2-T1* | T2-T1** | T1^$^  (SD) | T2-T1* | T2-T1** |
|  | n | 575 |  |  | 569 |  |  | 567 |  |  | 599 |  |  | 593 |  |  | 596 |  |  | 601 |  |  |
| ∆ QL5^a^ | QL deterioration | 73.3  (18.9) | −4.0 | 0.8 | 72.4  (20.2) | −6.8 | −5.5 | 67.8 (24.2) | −12.7 | −5.6 | 67.3 (25.1) | −9.3 | −5.1 | 74.7 (19.9) | −4.9 | −1.9 | 72.3  (19.2) | −2.3 | −1.4 | 59.5  (26) | 1.3 | −0.2 |
|  | QL  stability | 72.2  (19.1) | −1.0 | 4.1 | 70.2  (19.8) | −2.8 | −2.3 | 65.9 (23.3) | −8.4 | −2.0 | 63.5 (23.9) | −5.3 | −1.1 | 72.3 (20.4) | −1.6 | 0.5 | 71.2  (19.9) | 0.1 | −0.8 | 59.5  (25.8) | 3.9 | 1.4 |
|  | QL improvement | 71.5  (19.4) | 4.9 | 9.2 | 72.4  (20.2) | −2.5 | −1.0 | 68.3 (23.3) | −7.5 | −0.3 | 64.8 (24.3) | −2.0 | 1.3 | 70.9 (20.9) | −0.7 | 2.0 | 70.8  (20.0) | 2.0 | 1.8 | 56.2  (26.3) | 7.9 | 6.5 |
|  | p-value |  | <0.0001 | 0.0002 |  | 0.04 | 0.05 |  | 0.07 | 0.07 |  | 0.02 | 0.04 |  | 0.1 | 0.14 |  | 0.1 | 0.22 |  | 0.05 | 0.04 |
| ∆ QL10^b^ | QL deterioration | 73.2  (18.1) | −4.5 | −0.5 | 72.3  (20.3) | −7.5 | −6.6 | 68.1 (24.4) | −13.1 | −6.8 | 67.8 (24.3) | −9.2 | −5.9 | 76.7 (19.1) | −7.0 | −5.7 | 74.3  (18.4) | −3.8 | −4.3 | 61.0  (25.5) | 0.6 | −2.0 |
|  | QL  stability | 72.0  (19.5) | 0.3 | 4.5 | 71.0  (19.9) | −2.3 | −2.5 | 66.1 (23.4) | −8.8 | −2.1 | 64.0 (24.7) | −4.7 | −1.1 | 70.7 (20.7) | 2.4 | 2.7 | 69.8  (20.1) | 3.2 | 1.0 | 57.3  (26.0) | 5.7 | 2.5 |
|  | QL improvement | 72.8  (19.5) | 8.1 | 11.6 | 72.8  (20.5) | −0.4 | 0.3 | 70.0 (23.2) | −5.0 | 0.8 | 65.6 (24.1) | −0.8 | 1.8 | 72.9 (20.6) | 1.5 | 1.5 | 72.0  (19.9) | 3.7 | 2.6 | 58.1  (26.8) | 10.0 | 8.5 |
|  | p-value |  | <0.0001 | <0.0001 |  | 0.006 | 0.01 |  | 0.03 | 0.03 |  | 0.03 | 0.03 |  | <0.0001 | <0.0001 |  | 0.0003 | 0.001 |  | 0.02 | 0.004 |
| Mean differences of Sat35 (period T1-T3) | | SATGEN | | | SATDTS | | | SATDIS | | | SATDIP | | | SATNTS | | | STANIS | | | SATNIP | | |
|  |  | T1^$^  (SD) | T3-T1* | T3-T1** | T1^$^  (SD) | T3-T1* | T3-T1** | T1^$^  (SD) | T3-T1* | T3-T1** | T1^$^  (SD) | T3-T1* | T3-T1** | T1^$^  (SD) | T3-T1* | T3-T1** | T1^$^  (SD) | T3-T1* | T3-T1** | T1^$^  (SD) | T3-T1* | T3-T1** |
|  | n | 572 |  |  | 575 |  |  | 574 |  |  | 572 |  |  | 569 |  |  | 571 |  |  | 577 |  |  |
| ∆ QL5^a^ | QL deterioration | 74.4  (20.2) | −2.7 | −0.5 | 73.3  (19.3) | −13.5 | −8.1 | 69.9 (22.3) | −16.0 | −8.3 | 67.1 (23.8) | −8.2 | −7.0 | 75.4 (19.6) | −11.1 | −6.1 | 73.7  (18.8) | −8.8 | −8.0 | 61.4  (25.4) | −5.2 | −6.8 |
|  | QL  stability | 75  (19) | −0.5 | 1.8 | 73.6  (20) | −8.0 | −2.8 | 68.6 (24.5) | −7.9 | −0.3 | 66.8 (25.6) | −0.8 | −0.3 | 74.1 (21.1) | −5.1 | −0.2 | 73.2  (20.6) | −1.9 | −1.3 | 60.3  (26.6) | 4.1 | 2.0 |
|  | QL improvement | 69.3  (18.9) | 4.6 | 6.8 | 69.6  (20.9) | −3.8 | 1.3 | 64.4 (24.1) | −4.4 | 2.9 | 63.9  (25) | 3.4 | 3.6 | 70.2 (20.9) | −5.3 | −0.3 | 69.1  (20.1) | −1.7 | −1.0 | 56.4  (25.5) | 3.9 | 1.4 |
|  | p-value |  | 0.0025 | 0.002 |  | <0.0001 | <0.0001 |  | <0.0001 | <0.0001 |  | <0.0001 | 0.0001 |  | 0.01 | 0.01 |  | 0.0005 | 0.0006 |  | 0.0007 | 0.002 |
| ∆ QL10^b^ | QL deterioration | 74.8  (21.2) | −3.0 | −1.2 | 72.4  (20) | −14.0 | −9.3 | 68.6 (23.4) | −15.7 | −8.7 | 66  (24.8) | −9.4 | −8.8 | 75.1 (20) | −10.6 | −6.0 | 73.2  (19.9) | −8.4 | −7.9 | 61.9  (25.3) | −6.5 | −8.6 |
|  | QL  stability | 73.6  (19.1) | 0.3 | 2.4 | 73.3  (19.9) | −8.0 | −3.3 | 68.7 (23.7) | −9.0 | −2.0 | 67.6 (24.7) | −0.8 | −1.3 | 73.7 (20.5) | −7.0 | −2.4 | 72.7  (19.6) | −4.0 | −3.7 | 60  (25.9) | 3.0 | −0.1 |
|  | QL improvement | 68.8  (18.5) | 5.4 | 7.3 | 69  (20.8) | −2.4 | 2.4 | 63.7 (23.9) | −2.5 | 4.3 | 62.1  (24.8) | 5.7 | 5.5 | 69.7 (21.1) | −3.9 | 0.9 | 68.8  (20.4) | −0.2 | 0.4 | 55.2  (25.7) | 5.7 | 2.7 |
|  | p-value |  | 0.005 | 0.004 |  | <0.0001 | <0.0001 |  | 0.0001 | 0.0001 |  | <0.0001 | <0.0001 |  | 0.06 | 0.05 |  | 0.005 | 0.004 |  | 0.0005 | 0.001 |

Numbers shown in bold are significant p-values. T1 : start of treatment, T2 : end of treatment, T3 : three months after the end of treatment.

^$^ crude mean score at T1 assessment by category of global health changes.

* Satisfaction scores differences in the model localization adjusted for age, marital status, centre and level of global health at inclusion.

** Satisfaction scores differences in the model radiotherapy adjusted for age, marital status, centre and level of global health at inclusion.

^a^ minimal difference scores of 5 points for global health changes: the percentage of patients in the 3 categories, deterioration, stability and improvement, was 41%, 31% and 28% at T2, and 30%, 28%, 42% at T3 respectively.

^b^ minimal difference scores of 10 points for global health changes: the percentage of patients in the 3 categories, deterioration, stability and improvement, was 30%, 52% and 18% at T2, and 21%,50% and 29% at T3 respectively.
