# Supplementary material for: A longitudinal analysis of patient satisfaction with care and quality of life in ambulatory oncology based on the OUT-PATSAT35 questionnaire
Source: BMC Cancer. 2014 Jan 25;14:42. doi: 10.1186/1471-2407-14-42 (PMC3922727; doi:10.1186/1471-2407-14-42)
Supplement: Additional file 2 — Cross-sectional analysis of satisfaction scores at T2 and T3 assessments by QoL changes. [file 1471-2407-14-42-S2.doc]

**Additional file 2:** Cross-sectional analysis of satisfaction scores at T2 and T3 assessments by QoL changes

| Satisfaction scores at T2 | | | SATGEN | | SATDTS | | SATDIS | | SATDIP | | SATDAV | | SATNIS | | SATNIP | | SATNAV | | SATEXE | | SATWAI | | SATPE | |
| --- | --- | --- | --- | --- | --- | --- | --- | --- | --- | --- | --- | --- | --- | --- | --- | --- | --- | --- | --- | --- | --- | --- | --- | --- |
|  |  |  | Loc | Radio | Loc | Radio | Loc | Radio | Loc | Radio | Loc | Radio | Loc | Radio | Loc | Radio | Loc | Radio | Loc | Radio | Loc | Radio | Loc | Radio |
|  | QL  deterioration | mean Sat35 score* | 62.4 | 71.9 | 56.3 | 67.2 | 52.2 | 62.3 | 43.8 | 60.2 | 43.9 | 59.5 | 62.6 | 70.1 | 44.6 | 58.4 | 53.7 | 63.6 | 52.7 | 62.4 | 55.6 | 60.7 | 50.4 | 57.8 |
| ∆ QL5^a^ | QL  stability | mean difference | 4.5 | 4.0 | 5.1 | 4.6 | 5.7 | 5.4 | 5.1 | 4.8 | 4.7 | 3.3 | 2.8 | 1.7 | 5.6 | 5.5 | 3.7 | 3.5 | 5.2 | 4.4 | 2.3 | 2.3 | 4.1 | 3.0 |
|  | QL  improvement | mean difference | 12.9 | 12.6 | 12.1 | 11.7 | 13.7 | 13.1 | 13.9 | 12.1 | 9.0 | 7.8 | 10.1 | 7.5 | 14.5 | 11.8 | 12.6 | 10.9 | 11.8 | 10.4 | 11.2 | 7.7 | 9.2 | 9.1 |
|  |  | p-value | <0.0001 | <0.0001 | <0.0001 | <0.0001 | <0.0001 | <0.0001 | 0.0001 | 0.001 | 0.0004 | 0.007 | 0.0004 | 0.01 | 0.0001 | 0.004 | 0.0001 | 0.001 | 0.0005 | 0.0008 | 0.001 | 0.005 | 0.0003 | 0.0002 |
|  | QL deterioration | mean Sat35 score* | 62.1 | 70.2 | 55.7 | 65.5 | 51.7 | 60.9 | 44.6 | 59.5 | 43.5 | 58.4 | 62.0 | 68.8 | 44.5 | 57.6 | 53.0 | 62.0 | 52.7 | 62.6 | 47.7 | 60.3 | 50.9 | 57.9 |
| ∆ QL10^b^ | QL  stability | mean difference | 5.8 | 6.2 | 7.3 | 6.7 | 6.2 | 6.6 | 4.3 | 5.6 | 4.5 | 4.4 | 4.8 | 3.6 | 5.9 | 5.4 | 6.1 | 5.4 | 5.2 | 3.4 | 1.1 | 2.6 | 2.3 | 2.7 |
|  | QL  improvement | mean difference | 18.5 | 18.9 | 17.3 | 16.5 | 20.3 | 18.9 | 16.3 | 15.4 | 13.8 | 11.6 | 13.3 | 11.7 | 18.3 | 16.8 | 18.6 | 16.8 | 11.8 | 12.9 | 12.3 | 11.8 | 11.8 | 11.3 |
|  |  | p-value | <0.0001 | <0.0001 | <0.0001 | <0.0001 | <0.0001 | <0.0001 | <0.0001 | 0.0004 | <0.0001 | 0.0008 | 0.0002 | 0.001 | 0.0001 | 0.001 | <0.0001 | <0.0001 | 0.0006 | 0.0009 | <0.0001 | 0.0002 | <0.0001 | 0.0003 |
| Satisfaction scores at T3 | | | SATGEN |  | SATDTS |  | SATDIS |  | SATDIP |  | SATDAV |  | SATNIS |  | SATNIP |  | SATNAV |  | SATEXE |  | SATWAI |  | SATPE |  |
|  |  |  | Loc | Radio | Loc | Radio | Loc | Radio | Loc | Radio | Loc | Radio | Loc | Radio | Loc | Radio | Loc | Radio | Loc | Radio | Loc | Radio | Loc | Radio |
|  | QL  deterioration | mean Sat35 score* | 65.8 | 72.3 | 55.9 | 66.0 | 54.4 | 61.4 | 47.0 | 57.6 | 44.2 | 53.4 | 59.6 | 66.2 | 47.5 | 54.6 | 51.8 | 58.7 | 54.8 | 60.2 | 50.2 | 57.3 | 45.6 | 57.8 |
| ∆ QL5^a^ | QL  stability | mean difference | 3.1 | 3.3 | 5.3 | 5.5 | 8.0 | 7.9 | 7.2 | 7.6 | 7.8 | 7.9 | 6.0 | 6.0 | 8.4 | 8.9 | 7.2 | 7.4 | 5.5 | 5.7 | 9.6 | 9.9 | 4.5 | 5.1 |
|  | QL  improvement | mean difference | 6.5 | 6.4 | 10.9 | 10.6 | 12.4 | 11.9 | 14.0 | 14.0 | 9.9 | 9.5 | 7.0 | 6.5 | 8.9 | 8.9 | 9.6 | 9.6 | 7.5 | 7.4 | 7.0 | 6.7 | 3.3 | 3.4 |
|  |  | p-value | 0.32 | 0.34 | 0.009 | 0.01 | 0.02 | 0.02 | 0.002 | 0.0007 | 0.01 | 0.01 | 0.19 | 0.24 | 0.17 | 0.1 | 0.005 | 0.004 | 0.4 | 0.34 | 0.03 | 0.02 | 0.35 | 0.26 |
|  | QL  deterioration | mean Sat35 score* | 64.9 | 70.8 | 54.4 | 63.6 | 53.2 | 59.1 | 44.8 | 54.0 | 43.3 | 51.7 | 59.1 | 65.2 | 46.6 | 52.9 | 51.4 | 57.3 | 54.6 | 59.4 | 50.6 | 57.0 | 44.0 | 55.3 |
| ∆ QL10^b^ | QL  stability | mean difference | 4.1 | 4.5 | 7.4 | 7.9 | 8.9 | 9.3 | 10.8 | 11.5 | 7.6 | 7.8 | 5.0 | 4.9 | 7.7 | 8.5 | 6.8 | 7.1 | 4.3 | 4.8 | 5.8 | 6.4 | 5.2 | 6.0 |
|  | QL  improvement | mean difference | 9.1 | 9.0 | 14.7 | 14.6 | 16.7 | 16.4 | 19.0 | 19.2 | 13.3 | 13.2 | 10.1 | 9.7 | 12.0 | 12.1 | 12.7 | 12.8 | 10.0 | 10.0 | 8.8 | 8.7 | 7.5 | 7.9 |
|  |  | p-value | 0.12 | 0.13 | 0.001 | 0.001 | 0.002 | 0.002 | 0.0002 | <0.0001 | 0.002 | 0.002 | 0.04 | 0.06 | 0.15 | 0.1 | 0.0025 | 0.002 | 0.17 | 0.15 | 0.02 | 0.02 | 0.02 | 0.01 |

Numbers shown in bold are significant p-values. T1 : start of treatment, T2 : end of treatment, T3 : three months after the end of treatment.

^a^ minimal difference scores of 5 points for global health changes: the percentage of patients in the 3 categories, deterioration, stability and improvement, was 41%, 31% and 28% at T2, and 30%, 28%, 42% at T3 respectively.

^b^ minimal difference scores of 10 points for global health changes: the percentage of patients in the 3 categories, deterioration, stability and improvement, as 30%, 52% and 18% at T2, and 21%,50% and 29% at T3 respectively.

* reference class is deterioration of global health.

Abbreviations: QL = Global health, SATGEN = overall satisfaction, SATDTS doctors’technical skills, SATDIS = doctors’interpersonal skills, SATDIP = doctors’ information provision, SATDAV doctors’availability, SATNIS = nurses’interpersonal skills SATNIP = nurses’information provision, SATNAV = nurses’availability, SATEXE = exchange of information between caregivers, SATWAI = waiting-time, SATPE = physical environment.

Loc = Satisfaction scores in the model localization adjusted forage, marital status, centre and level of global health at inclusion.

Radio = Satisfaction scores differences in the model radiotherapy adjusted for age, marital status, centre and level of global health at inclusion.
